# Patient with multiple acyl-CoA dehydrogenase deficiency disease and ETFDH mutations benefits from riboflavin therapy: a case report

**DOI:** 10.1186/s12920-018-0356-8

**Published:** 2018-04-03

**Authors:** Liuh Ling Goh, Yingshan Lee, Ee Shien Tan, James Soon Chuan Lim, Chia Wei Lim, Rinkoo Dalan

**Affiliations:** 1 Molecular Diagnostic Laboratory, Tan Tock Seng Hospital, 11 Jalan Tan Tock Seng, Singapore, 308433 Singapore; 2 Department of Endocrinology, Tan Tock Seng Hospital, 11 Jalan Tan Tock Seng, Singapore, 308433 Singapore; 3 Department of Paediatrics, Genetics Services, KK Women’s and Children’s Hospital, 100 Bukit Timah Road, Singapore, 229899 Singapore; 4 Biochemical Genetics and National Expanded Newborn Screening, Department of Pathology and Laboratory Medicine, KK Women’s and Children’s Hospital, 100 Bukit Timah Road, Singapore, 229899 Singapore; 5 Lee Kong Chian School of Medicine, Nanyang Technological University, 11 Mandalay Road, Singapore, 308232 Singapore; 6 Yong Loo Lin School of Medicine, National University of Singapore, 12 Science Drive 2, Singapore, 117549 Singapore

**Keywords:** *ETFDH*, Lipid storage myopathy, Multiple acyl-CoA dehydrogenase deficiency, Whole exome sequencing

## Abstract

**Background:**

Lipid storage myopathy (LSM) is a diverse group of lipid metabolic disorders with great variations in the clinical phenotype and age of onset. Classical multiple acyl-CoA dehydrogenase deficiency (MADD) is known to occur secondary to mutations in electron transfer flavoprotein dehydrogenase (ETFDH) gene. Whole exome sequencing (WES) with clinical correlations can be useful in identifying genomic alterations for targeted therapy.

**Case presentation:**

We report a patient presented with severe muscle weakness and exercise intolerance, suggestive of LSM. Diagnostic testing demonstrated lipid accumulation in muscle fibres and elevated plasma acyl carnitine levels. Exome sequencing of the proband and two of his unaffected siblings revealed compound heterozygous mutations, c.250G > A (p.Ala84Thr) and c.770A > G (p.Tyr257Cys) in the *ETFDH* gene as the probable causative mutations. In addition, a previously unreported variant c.1042C > T (p.Arg348Trp) in *ACOT11* gene was found. This missense variant was predicted to be deleterious but its association with lipid storage in muscle is unclear. The diagnosis of MADD was established and the patient was treated with riboflavin which resulted in rapid clinical and biochemical improvement.

**Conclusions:**

Our findings support the role of WES as an effective tool in the diagnosis of highly heterogeneous disease and this has important implications in the therapeutic strategy of LSM treatment.

**Electronic supplementary material:**

The online version of this article (10.1186/s12920-018-0356-8) contains supplementary material, which is available to authorized users.

## Background

Lipid storage myopathy (LSM) is a diverse group of lipid metabolic disorders characterized by impaired fatty acids oxidation [1]. Multiple acyl-coenzyme A dehydrogenase deficiency (MADD) is an autosomal recessive disorder characterized by mitochondrial electron transfer system defects and impaired fatty acids metabolism [2]. MADD is associated with a highly diverse clinical phenotype, ranging from the lethal neonatal onset type with congenital anomalies, to the adult onset type with milder and variable clinical presentation [3]. The clinical heterogeneity of adult-onset forms pose a challenge for diagnosis with the vast majority displaying mild or atypical biochemical abnormalities [4]. Diagnosis of MADD is aided by urinary organic acid analysis and blood acylcarnitine profile.

Classical MADD has been known to occur secondary to deficiency in electron transfer flavoprotein (ETF) or ETF:ubiquinone oxidoreductase (ETF:QO). The severity of the disease is dependent on the location and nature of mutations in the genes encoding ETF or ETF:QO. Null mutations result in a complete loss of protein expression or function leading to lethal disease. Missense mutations result in only a partial loss of enzyme activity, with preservation of some residual enzyme activity thus leading to a milder clinical phenotype [5–7].

Some MADD patients have an improvement in the clinical symptoms and metabolic profile with riboflavin treatment. These patients, referred to as riboflavin-responsive MADD (RR-MADD) patients, have been seen to harbour variations in the *ETFDH* gene encoding ETF:QO. To date, more than 50 different *ETFDH* variations have been reported in RR-MADD [8–12]. The ETF:QO protein consists of a flavin adenine dinucleotide (FAD) binding domain and a [4Fe-4S] cluster. This protein is responsible for ATP production by mediating the transfer of reducing equivalents to the respiratory ubiquinone pool [13, 14]. In RR-MADD, the missense mutations lead to a misfolding of protein leading to decreased protein stability [15–17]. Riboflavin is the substrate that is converted to FAD. Increasing the availability of riboflavin, is thus speculated to promote folding and/or stabilizes the native conformation of certain types of ETF:QO variant proteins.

Here, we report a 65 year old male individual with diabetes presenting severe muscle weakness and exercise intolerance as major symptoms. Whole exome sequencing (WES) revealed that he is a compound heterozygous for two variants in the *ETFDH* gene, establishing the final diagnosis and responded to riboflavin supplementation. The study also identified a previously unreported variant in *ACOT11* with highly-predicted functional impact.

## Case presentation

**Fig. 1:**
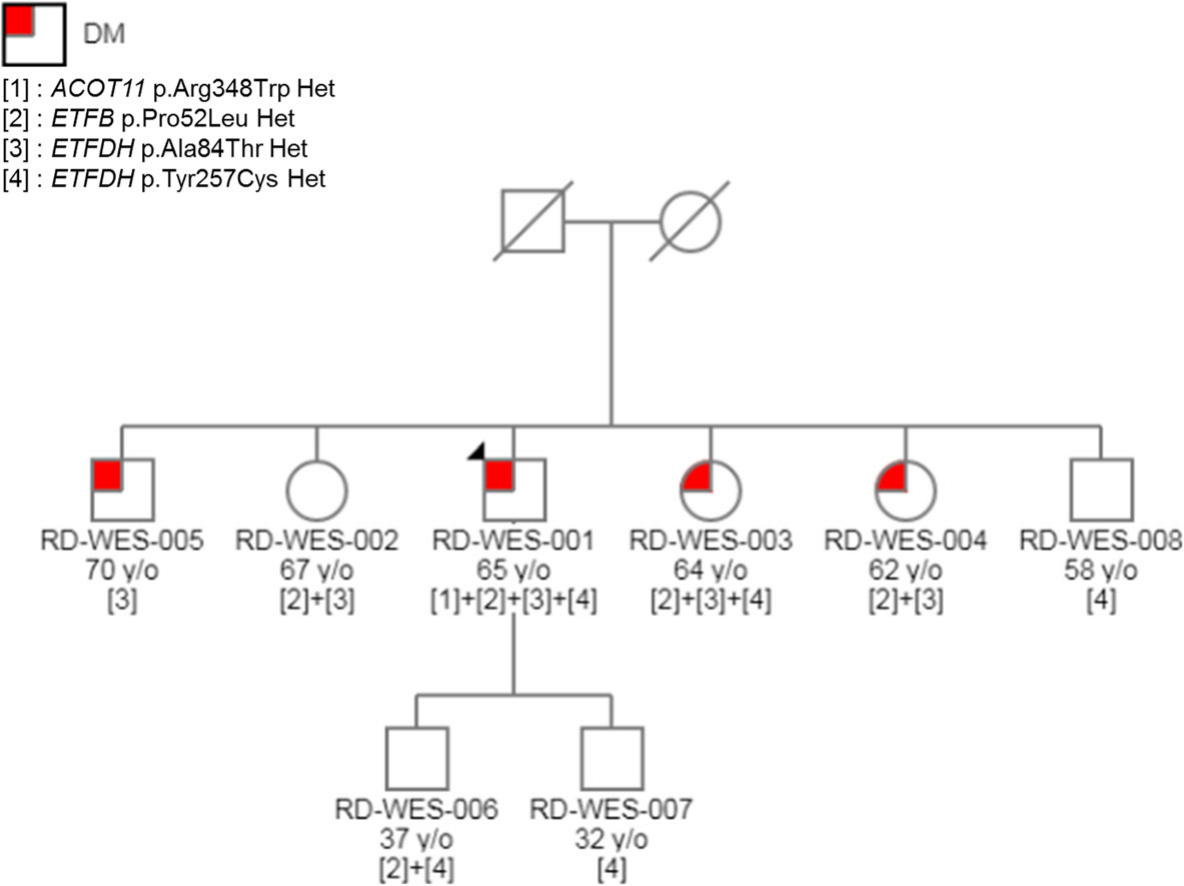
Pedigree showing co-segregation of the heterozygous changes within *ETFDH*, *ETFB* and *ACOT11* in the family. Open symbol represents unaffected subject. Patient (proband) is indicated with a black arrow

The proband was a 65 year old patient with a relapsing and remitting course of lipid storage myopathy. His symptoms started at 17 years old when he was enlisted into the military. He noted muscle fatigue and exercise intolerance affecting only his lower limbs after intense exercise. These symptoms recurred with greater severity at 36 years old. His upper and lower limbs, as well as his neck muscles were affected. He also experienced difficulty in swallowing. Possible aggravating factors were irregular meals and heavy alcohol consumption. He was evaluated in another centre at that time and we were able to access part of his results. Creatinine kinase levels were elevated 20 times. Muscle biopsy histopathology showed the presence of fat globules within vacuolated muscle fibres and increased oxidative enzyme activates, suggesting a ‘lipid storage disease’. He had received oral steroids intermittently, but no specific treatment was given as his symptoms resolved spontaneously. Recently, his symptoms recurred, and he was referred to our centre. In the past 2 years, he had picked up jogging and begun to experience similar episodes of muscle fatigue affecting only his lower limbs after exercise. Exercise intolerance had deteriorated and he can only manage 100 m of walking each time by the time he consulted our centre. No other muscles were affected. There were no accompanying cardiac or respiratory symptoms such as chest pain or breathlessness. His other medical problems included type 2 diabetes mellitus and hyperlipidemia. We noted he has a strong family history of type 2 diabetes mellitus, with 3 out of 5 siblings being inflicted with the disease. His elder son has multiple medical problems associated with obesity – hypertension, aortic aneurysm, obstructive sleep apnea and stroke. His younger son has bicuspid aortic valve. There is no family history of consanguineous marriage or muscle disorders. To the best of our knowledge, his siblings and children do not suffer from any of the symptoms experienced by the proband (Fig. 1). Specifically, his siblings (RD-WES-002, RD-WES-003, RD-WES-004, RD-WES-005, and RD-WES-008) were determined as healthy based on their clinical phenotypes and normal creatinine kinase values.

On examination, he weighed 86.6 kg and his body mass index was 28.2 kg/m^2^. There were no dysmorphic features. Cushingoid features were absent. Facial muscle weakness and ptosis were absent. Neck muscles were normal. There was no winging of both scapulae. Wasting of both quadriceps were noted. Muscle strength was reduced at the shoulders and the hips with manual muscle testing 4 out of 5 for both flexors and extensors. Distal muscle strength was normal for both upper and lower limbs (Table 1). Fatigability of the muscles was not demonstrated in the clinic. Muscle tone and reflexes were normal. He displayed normal gait. Examination of the cardiorespiratory and abdominal systems were normal.

**Table 1:** Manual muscle testing (MMT) scale of upper limbs and lower limbs of the proband on examination

	Neck		Shoulders		Elbows		Fingers	Hips		Knees		Ankles	
	Flexion	Extension	Abduction	Adduction	Flexion	Extension	Finger grip	Flexion	Extension	Flexion	Extension	Flexion	Extension
Power (out of 5)	5	5	4	5	5	5	5	4	4	5	5	5	5

Basic investigations revealed elevated values of creatinine kinase and myoglobin. Liver function tests, renal function tests and thyroid function tests were normal (Table 2). Tests specific to lipid storage disease that were performed included plasma acylcarnitines, serum carnitine level, serum riboflavin, urine acylglycines and urine organic acids. The results from the plasma acylcarnitines were abnormal, showing elevated concentrations of several acylcarnitine species (C5-C18) compared to reference range (Table 3).

**Table 2:** Laboratory results of the patient

Tests	Results	Reference index	Units
Albumin	38	35–48	g/L
Alanine transferase	37	17–63	U/L
Alkaline phosphatase	46	38–126	U/L
Bilirubin	12	7–31	umol/L
Calcium	2.28	2.15–2.58	mmol/L
Carnitine, serum			
Carnitine, total	47	34–78	nmol/ml
Carnitine, free (FC)	28	25–54	nmol/ml
Acylcarnitine (AC)	19	5–30	nmol/ml
AC/FC Ratio	0.7	0.1–0.8	
Creatinine	66	60–105	umol/L
Creatinine kinase	**492**	50–250	U/L
Glucose, fasting	6.0	3.0–6.0	mmol/L
Glycated Hemoglobin	6.4	–	%
Lipid Panel			
Cholesterol	5.2		mmol/L
HDL-C	1.4		mmol/L
LDL-C	3.4		mmol/L
Triglycerides	0.9		mmol/L
Myoglobin	**294**	16–96	ug/L
Riboflavin, serum	6	1–19	ug/L
Thyroxine, free	13	8–21	pmol/L
TSH	2.69	0.34–5.60	mIU/L
25-OH vitamin D	24	> 20	ug/L

Numbers out of reference index are in bold

**Table 3:** Acylcarnitine profile of the patient before and after riboflavin treatment

Analytes	Results		Reference range	Units
	Before	After		
C0- Free carnitine	36	42	17–64	umol/L
C2- Acetylcarnitine	10	3	2-17	umol/L
C3-Propionylcarnitine	0.42	0.31	0.14–0.95	umol/L
C3DC-Malonylcarnitine	0.03	0.03	0.01–0.08	umol/L
C4-n-butyryl−/isobutyrylcarnitine	0.33	0.16	0.08–0.46	umol/L
C4OH-3-Hydroxy-Butyrylcarnitine	0.12	0.06	0.01–0.24	umol/L
C5-Isovaleryl−/2-Methylbutyrylcarnitine	**0.39**	0.10	0.03–0.32	umol/L
C5:1-Tiglylcarnitine	0.02	0.01	0.01–0.04	umol/L
C5OH-3-Hydroxy-Isovaerylcarnitine	0.03	0.03	0.01–0.09	umol/L
C5DC-Glutaryl/3-Hydroxydecanoylcarnitine	**0.28**	0.06	0.010.08	umol/L
C6-Hexanoylcarnitine	**0.43**	**0.25**	0.02–0.12	umol/L
C8-Octanoylcarnitine	**1.50**	**1.69**	0.03–0.22	umol/L
C10-Decanoylcarnitine	**3.07**	**1.82**	0.05–0.42	umol/L
C10:1-Decenoylcarnitine	**0.40**	**0.61**	0.03–0.26	umol/L
C10:2-Decadienoylcarnitine	0.05	0.05	0.01–0.05	umol/L
C12-Dodecanoylcarnitine	**0.87**	**0.15**	0.02–0.13	umol/L
C12:1-Dodecenoylcarnitine	**0.13**	0.09	0.01–0.10	umol/L
C14-Tetradecanoylcarnitine	**0.51**	0.05	0.01–0.07	umol/L
C14:1-Tetradecenoylcarnitine	**0.72**	0.15	0.01–0.17	umol/L
C14:2-Tetradecadienoylcarnitine	**0.20**	**0.07**	0.01–0.05	umol/L
C16-Hexadecanoylcarnitine	**0.81**	0.13	0.06–0.24	umol/L
C16:1-Hexadecenoylcarnitine	**0.53**	0.05	0.01–0.07	umol/L
C18-Octadecanoylcarnitine	**0.34**	0.04	0.02–0.10	umol/L
C18:1-Octadecenoylcarnitine	**0.95**	0.12	0.05–0.28	umol/L
C18:2-Linoleylcarnitine	**0.33**	0.05	0.02–0.10	umol/L
C16OH-3-Hydroxy-Hexadecanoylcarnitine	0.02	0.01	0.00–0.02	umol/L

Numbers out of reference range are in bold

### Clinical assessment and blood collection

Clinical diagnosis was based on physical examination, muscle biopsy, biochemical tests. Peripheral blood samples were taken from the patient and his family members, including his 5 siblings and 2 children, aged 32 to 70. The study was according to the Declaration of Helsinki Principles and the ethical guidelines of our institution. Written informed consent was obtained prior to sample collection.

### Genetic testing

Genomic DNA was extracted from peripheral blood using QIAamp DNA kit (Qiagen). Whole exome library preparation was performed for patient (RD-WES-001) and two siblings (RD-WES-005 and RD-WES-008) using Nimblegen SeqCap EZ Library SR kit (Roche) according to manufacturer instruction and enriched samples underwent paired-end sequencing on Illumina HiSeq 4000 device (Macrogen, Korea). Candidate variants were validated and followed up in the proband and his relatives with Sanger-based sequencing. Primers used were shown in Additional file 1: Table S1.

The mapping of sequencing reads to NCBI GRCh37 human reference genome and variant detection were performed using the Burrows-Wheeler read aligner (BWA) and Genome Analysis Tool Kit (GATK), respectively. The detected single nucleotide polymorphisms (SNPs), deletion and insertion variants in coding regions and splice sites sequences were filtered by quality/depth, minimum total read depth (coverage). Variants passing these quality filters were annotated with information from public annotation databases using ANNOVAR. We exclude common variants with allele frequency greater than 0.05 in the 1000 Genomes and ExAC Browser. Choice of variants for further analysis were limited to genes implicated in glutaric academia, carnitine metabolism and acyl coenzyme metabolism; and supported by in silico analysis by different bioinformatics tools (PolyPhen2 and SIFT).

### Molecular findings

The sequencing generated 67,408,294 reads in the proband, 65, 560,602 in his older brother (RD-WES-005), and 18, 657, 150 in his younger brother (RD-WES-008). More than 99.9% of these reads were mapped and more than 74.7% were on-targets reads. Percentage of targets with more than 10X coverage in RD-WES-001, RD-WES-005 and RD-WES-008 were 95.5, 95.3 and 62.4% respectively; and their corresponding mean depth were 57.9X, 56.0X and 16.4X. The number of variants identified per sample ranged from 81,341 to 105, 643 (Additional file 1: Table S2). There was a total of 29,793 variants common across all 3 samples which may represent the genotypes shared among all the family members. These common variants were excluded from subsequent analysis, rendering a total of 3504 variants unique to the proband, 5986 variants common between proband and RD-WES-005, and 1500 variants common between proband and RD-WES-008. Although the specific mutations causing the myopathy syndrome are more likely to be found in set of patient specific mutations, there is a chance that causative mutations belong to the common variants shared with his siblings, if the patient is the only family member with the respective homozygous genotype. Among these, data annotation predicted 515 sample specific missense mutations in the proband, 314 missense variants common between proband and RD-WES-005, and 247 missense variants common between proband and RD-WES-008. No frameshift, insertion or deletion mutations were found. We further filtered these variants based on targeted pathways, minor allele frequency (MAF≤0.05 in 1000 Genome and ExAC databases) and predicted as damaging by two different computer algorithms, SIFT and PolyPhen2. As a result, we obtained a final list of 3 variants in the coding region of *ETFDH* and *ACOT11* genes (Table 4). Among these, *ETFDH* c.250G > A (Ala84Thr) was characterized in the ClinVar database, as pathogenic. *ETFDH* c.770A > G (Tyr257Cys) was not reported in 1000 Genome or ExAC databases but has been reported with *ETFDH* c.250G > A in riboflavin-responsive lipid storage myopathy [8]. Hence, *ETFDH* Ala84Thr and Tyr257Cys represent excellent candidates as causative event underlying the trait. *ACOT11* c.1042C > T (Arg348Trp) represents a missense variant that has not been reported in the literature in individuals with a LSM related disease. In silico analyses predict that this variant is probably damaging to protein structure and function.

**Table 4:** Number of candidate variants

Gene	SNP ID	Base change	1000 Genome					ExAC	Protein change	SIFT	POLYPhen	Class
			AFR	AMR	EAS	EUR	SAS					
*ETFDH*	rs121964954	250G > A	0	0	0.001	0	0	0.00173	Ala84Thr	Deleterious	Damaging	Pathogenic
*ETFDH*	–	770A > G	–	–	–	–	–	–	Tyr257Cys	Deleterious	Damaging	VUS
*ACOT11*	rs139751558	1042C > T	0	0	0.0198	0	0	0.04853	Arg384Trp	Deleterious	Damaging	VUS

**Fig. 2:**
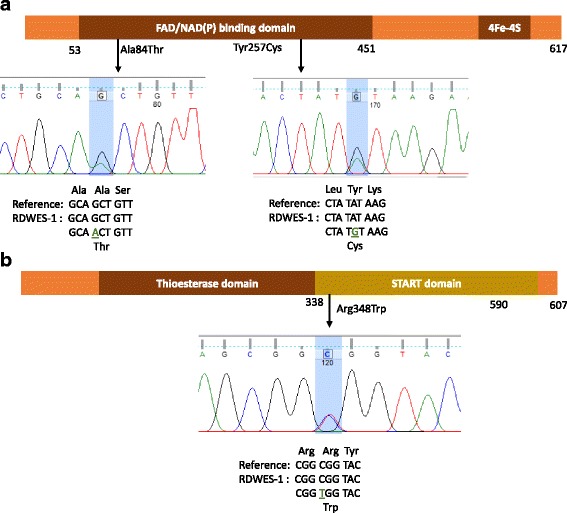
Electropherograms and locations of missense mutations in *ETFDH* (**a**) and *ACOT11* (**b**) sequences. Protein domains are schematized with numbers indicating the amino acid

All available members in the pedigree were screened for these three mutations. Sanger sequencing analysis validated the mutations in the proband (Fig. 2) and revealed that *ACOT11* c.1042C > T variant was unique to him. In addition, one of his healthy sister (RD-WES-003) harboured the same combination of *ETFDH* mutations. The other family members had only one of the heterozygous *ETFDH* mutations (data not shown).

### Structural analysis

The 3-dimensional structure of the human ETF:QO protein was predicted using the SWISS-MODEL protein structure homology modelling server to evaluate the possible consequences of the missense mutation [18]. The porcine ETF:QO (PDB entry: 2GMH) shared a high amino acid sequence similarity with the human homolog and was as the model template [13]. The program DeepView (Swiss Pdb viewer) was used for visualization and analysis of the modelled protein structure.

**Fig. 3:**
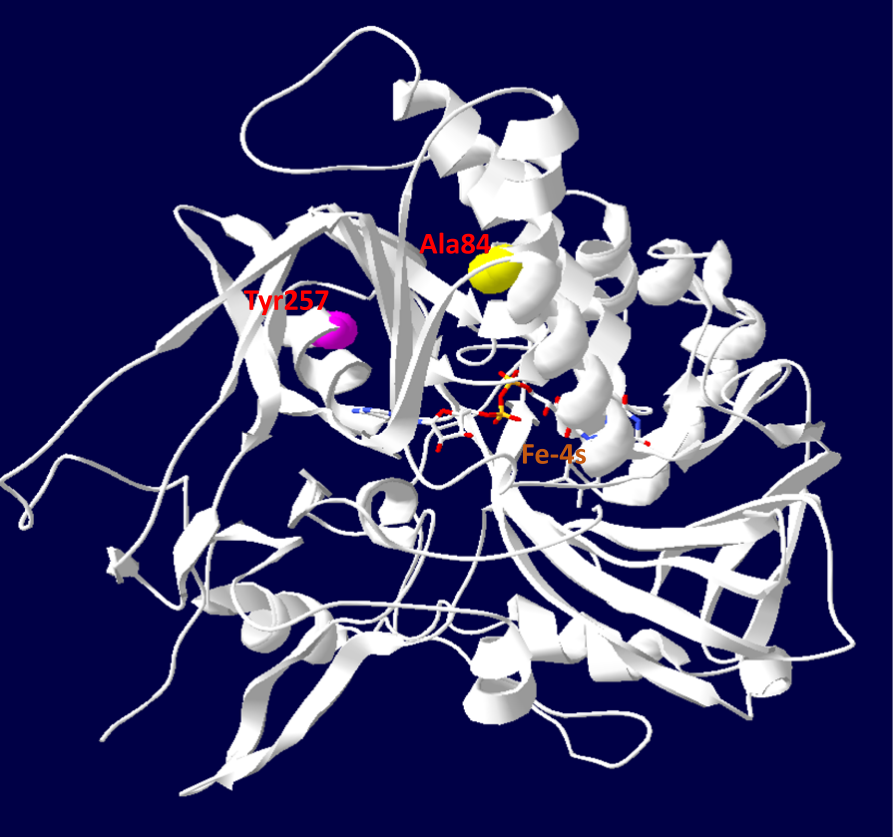
Structure prediction of ETF:QO. The predicted wild-type model of human ETF:QO with Ala84 and Tyr257 in the hydrophobic FAD-binding domain shown in ball-and-stick representations

Structural analysis of ETF:QO suggests that the wild-type Ala84 located within the FAD binding domain and the replacement with threonine disrupted the stability of FAD binding, which is essential for the activation of ETF:QO [19]. To explore the impact of Ala84Thr and Tyr257Cys on protein function, human ETF:QO model was constructed using the published porcine ETF:QO crystal structure (PDB Id:2GMH) as the template (Fig. 3). Both Ala84 and Tyr257 are hydrophobic residues located in helices within the FAD binding domain. The newly introduced mutant residues differ in size and hydrophobicity values from the wild-type residues. These differences are predicted to disturb the protein conformation and hence binding properties of the FAD binding domain.

### Treatment

The patient was given riboflavin 100 mg thrice daily with significant improvement in symptoms. He also received dietary counseling for a low fat diet with advice against prolonged fasting. Clinical improvement was supported by normalization of serum creatinine kinase and myoglobin levels, as well as improvement in plasma long chain acyl carnitine results (Table 3).

## Discussion and conclusions

In this report, we describe WES analysis of a patient with clinical features correspond to lipid storage myopathy and his healthy family members. Two compound heterozygous mutations in *ETFDH* gene were identified as the causative mutations. Within the *ETFDH* gene, the known pathogenic c.250G > A mutation was observed in homozygous and compound heterozygous state in several patients with MADD [8–10]. This variant (rs121964954) is more prevalent in Asians and reported in ExAC database with an allele frequency of 0.17% in East Asians. It has been reported in late-onset MADD patients from Asian countries including Taiwan, Hong Kong, Japan, Thailand and southern China [10–12, 20, 21]. To date, this mutation has never been reported in Western countries [9]. The other variant *ETFDH* c.770A > G was not present in ExAC database but reported in Chinese patients with MADD [22]. The study reported that the c.250G > A was more prevalent in Southern China, whereas c.770A > G had a higher frequency in Mainland China. Of note, *ETFDH* c.250G > A and c.770A > G mutations were also present in his healthy sister (RD-WES-003) who was 64 year old. Hence, RD-WES-003 may develop the symptoms associated with MADD in the future although the likelihoods are not high given her advance age. It is also possible that the compound heterozygous mutations in *ETFDH* can be tolerated or other genetic factors are required to cause disease.

Consistent with previous reports, supplementation of riboflavin, the precursor of FAD has strikingly improved the symptoms in this patient [4, 8, 23, 24]. FAD is a cofactor for ETF:QO which is important for the enzyme catalytic activity, correct folding, assembly and protein stability [25]. The ETF:QO Ala84Thr and Tyr257Cys mutations are located in the FAD binding domain. According to the predicted ETF:QO 3D structure, these residues are buried within the core of the protein. Alteration at these positions represent replacement of hydrophobic residues (alanine and tyrosine) with polar residues (threonine and cysteine). These drastic changes are likely to alter the conformation proximate to the FAD-binding site and disrupt the stability of FAD binding, which is essential for enzyme activation. Although the mechanism of riboflavin efficacy in MADD patients is not yet completely clear, a likely explanation is that of riboflavin increases the intra-mitochondrial FAD concentration and enhances the conformational stabilization of the mutant ETF:QO protein. In turn, this could ameliorate the effect of the mutations that reduce the affinity of ETF:QO for FAD [9, 26].

ACOT11 has been implicated in regulating the oxidation of fatty acids [27]. The *ACOT11* c.1042C > T variant (rs139751558) is ethnic-specific according to ExAC database, with an allele frequency of 4.85% in East Asians. The Arg348Trp variant resides in the START domain, which is involved in lipid binding and is essential for optimum enzyme activity [28]. The substitution represents a drastic change from a hydrophilic and positively charged residue to a hydrophobic and uncharged residue, which might impact lipid binding and impair oxidation of fatty acids. Intriguingly, this is the only patient specific variant that is predicted to be deleterious. However, without additional functional and/or genetic data, the significance of the alteration for disease is uncertain.

Due to its wide variety of clinical symptoms, LSM is difficult to diagnose. For cases highly suggestive of MADD, genetic analysis of *ETFDH* gene is highly recommended as vast majority of patients carry mutations in this gene [29]. For LSM, a large number of genes are implicated and targeted screening using Sanger sequencing will be an expensive and tedious option. In this study, we employed WES for comprehensive genetic diagnosis and have successfully revealed the mutations in *ETFDH* gene as the causal variants, leading to the personalized therapeutic strategy of riboflavin supplementation.

## Additional file

Additional file 1: **Table S1.** Primers sequences used to amplify and sequence candidate genes. **Table S2.** Number of candidate variants filtered against dbSNP and 1000 Genome public databases. (DOCX 45 kb)

## Abbreviations

BWA: Burrows-Wheeler read aligner
ETF: Electron transfer flavoprotein
ETF:QO: ETF:ubiquinone oxidoreductase
FAD: Flavin adenine dinucleotide
GATK: Genome Analysis Tool Kit
LSM: Lipid storage myopathy
MADD: Multiple acyl-CoA dehydrogenase deficiency
PCD: Primary carnitine deficiency
RR-MADD: Riboflavin-responsive MADD
WES: Whole-exome sequencing
