# Supplementary material for: Patient with multiple acyl-CoA dehydrogenase deficiency disease and ETFDH mutations benefits from riboflavin therapy: a case report
Source: BMC Med Genomics. 2018 Apr 3;11:37. doi: 10.1186/s12920-018-0356-8 (PMC5883299; doi:10.1186/s12920-018-0356-8)
Supplement: Supplementary file 1 — Table S1. Primers sequences used to amplify and sequence candidate genes. Table S2. Number of candidate variants filtered against dbSNP and 1000 Genome public databases. (DOCX 45 kb) [file 12920_2018_356_MOESM1_ESM.docx]

**Table S1**. Primers sequences used to amplify and sequence candidate genes.

| **Gene variant** | **Primer** | **Sequence (5’-3’)** | **Amplicon Length (bp)** |
| --- | --- | --- | --- |
| *ETFDH* c.250G>A | PRI-80F | CCCAGGAGTGAACATGGAAAGG | 162 |
|  | PRI-81R | GCTCCTATCTGGGCAGCTTTCT |  |
| *ETFDH* c.770A>G | PRI-82F | CATTTGCACTGTCTCTTCTACATCTG | 258 |
|  | PRI-83R | TCCTTCAGTCCAATCCCGTAGG |  |
| *ACOT11* c.1042C>T | PRI-84F | GTTTTGATTCCAGCTGCCCCAG | 228 |
|  | PRI-85R | GAATCCACATTCTGCCCACCAC |  |

**Table S2**. Number of candidate variants filtered against dbSNP and 1000 Genome public databases.

| **Sample** | **Number of SNPs** | **Number of % Found in dbSNP142** | **Number of synonymous SNPs** | **Number of indels** | **Number of Frameshift Variant** | **Number of Missense Variant** | **Number of Stop Gained** | **Number of Stop Lost** | **Ts/Tv Ratio** |
| --- | --- | --- | --- | --- | --- | --- | --- | --- | --- |
| RD-WES_1 | 105,517 | 95.6 | 11,755 | 15,528 | 311 | 11,084 | 107 | 38 | 2.2 |
| RD-WES_5 | 105,643 | 95.6 | 11,776 | 15,390 | 333 | 10,987 | 119 | 39 | 2.2 |
| RD-WES_8 | 81,341 | 96.6 | 10,164 | 9,738 | 252 | 9,396 | 89 | 35 | 2.3 |
